# Using WeChat official accounts to improve malaria health literacy among Chinese expatriates in Niger: an intervention study

**DOI:** 10.1186/s12936-016-1621-y

**Published:** 2016-11-24

**Authors:** Wei Li, Le Qiang Han, Yan Jun Guo, Jing Sun

**Affiliations:** 1 Health Care Center Manager of HSE Department, China National Oil and Gas Exploration and Development Corporation, 6-1 Fuchengmen Beidajie, Xicheng District, Beijing, 100034 China; 2 Infectious Disease Department, Dalian Sixth People’s Hospital, No. 269 Huibai Road, Lugang, Ganjingzi District, Dalian, 116031 Liaoning China; 3 Beijing Jishuitan Hospital, No. 31 Xinjiekou Street, Xicheng District, Beijing, 100035 China; 4 School of Nursing, Peking University Health Science Center, No. 38 Xueyuan Road, Haidian District, Beijing, 100191 China

**Keywords:** Malaria health literacy, WeChat official account, Chinese expatriates, Niger

## Abstract

**Background:**

Malaria is the main health risk for Chinese expatriates working in Niger. Health education is a recommended intervention for prevention of malaria among non-immune travellers and expatriate workers. It is urgent to develop an effective and feasible way for these populations to obtain information about the prevention and treatment of malaria.

**Methods:**

An individually randomized, unblinded, controlled trial was used to evaluate the effectiveness of using WeChat official accounts for health education to improve malaria health literacy among Chinese expatriates in Niger. A total 1441 participants completed a baseline malaria health literacy questionnaire and were randomly assigned to an intervention or comparison group in a ratio of 1:1. From July to October 2014, 50 malaria prevention and treatment messages were sent to the intervention group; 50 health news messages were concurrently sent to the control group. Both groups completed the malaria health literacy questionnaire again 4 months after the start of the education intervention. A questionnaire addressing satisfaction with the health education programme was completed by the intervention group. Malaria morbidity data for 2013 and 2014 were also collected.

**Results:**

At baseline, participant health literacy rates were 58.29, 62, 54, and 34% for skills, knowledge, practice, and attitude, respectively. After the intervention, rates for all four aspects of malaria literacy were above 70%. There was greater change in knowledge, attitude, practice, skills, and overall health literacy among the intervention group compared with the controls, with a statistically significant difference (*p* < 0.01). This was especially true for acquisition of malaria-related knowledge, practice and attitude; comprehensive intervention practices; and, correct use of rapid diagnostic tests (*p* < 0.001). The reported malaria morbidity during the study period decreased from 23.72 to 15.40%. Participants reported high levels of satisfaction with the WeChat health education programme with over 80% stating that they would continue to follow the programme.

**Conclusions:**

The present health education intervention, via a WeChat official account, for the prevention and treatment of malaria among non-immune travellers and expatriate workers proved to be an effective, sustainable, feasible, and well accepted strategy for improving malaria health literacy among Chinese expatriates in Niger.

## Background

According to the most recent World Health Organization (WHO) World Malaria Report, an estimated 214 million cases of malaria will occur in 2015, leading to 438,000 deaths. More than 80% of these cases and 90% of deaths occur in sub-Saharan Africa. Despite impressive decline in the number of malaria cases and deaths from 2000 to 2015, the population at risk of malaria has increased by 31% globally [1]. Malaria continues to be a significant global public health concern, particularly in sub-Saharan Africa. In Niger, malaria represents a substantial public health challenge and is the leading cause of morbidity and mortality [2]. Non-immune individuals include members of mobile populations, such as expatriate workers and travellers, who have no long-term exposure and thus lack efficacious protection against malaria. These populations are at considerably higher risk than endemic populations of contracting malaria, developing severe disease or dying [3]. Specific circumstances should be considered and special measures taken to protect these population groups from malaria infection [4]. Over the last ten years, Chinese outbound travel and export of labour services have grown dramatically. More than 50,000 Chinese expatriates work in Africa each year, and this number is rapidly increasing [5]. A health issue of great concern is that Chinese workers, having returned from Africa, represent most cases of imported malaria. A total of 37 African countries have been identified as the sources of imported cases, with most cases acquired in Ghana (9.9%), Equatorial Guinea (9.6%) and Nigeria (8.1%) [6–8]. Malaria has become the most important health issue for many international companies and the main factor influencing foreign workers’ regional selection decisions. Effective malaria health management and control strategies are required to reduce malaria morbidity among expatriate labourers.

Malaria is an entirely preventable, treatable and curable mosquito-borne disease. However, malaria morbidity among foreign workers in Niger remains high, with no visible trend towards being controlled [8]. At present, malaria represents more than a health management problem; the disease is increasingly recognized as an issue that can transcend medical support systems of international companies and significantly affect environmental, safety and human resource activities. The management of malaria is therefore a potential concern throughout the supply chain of global petroleum exploration, production, refining, distribution, and marketing [9]. Thus, examination is required of strategies for the prevention and treatment of malaria among non-immune expatriates travelling to and working in malaria-risk areas. The WHO recommends that workers who plan to travel or reside in areas of potential malaria risk should be provided with standardized educational materials and instruction prior to departure from their home countries [1]. To help eliminate malaria risk, residents in target areas must have a high level of knowledge regarding transmission and prevention of the disease and must maintain a high level of participation in activities related to this [10]. The WHO Global Malaria Programme recognizes the importance of the education sector. The Oil & Gas Producers/International Petroleum Industry Environmental Conservation Association (OGP/IPIECA) guide emphasizes the importance of improving the knowledge level of staff, creating a supportive social environment, and the vital role that companies and health managers can play in the prevention and control of malaria among their employees by establishing target behaviours [9] and providing material and moral support to help overcome factors that might affect those behaviours [10].

Health education intervention has proven to be an effective strategy for disease prevention, especially for chronic and communicable diseases [11, 12]. An understanding of perspectives and practices is one of the essential components of a successful programme for the prevention and treatment of malaria among non-immune travellers and expatriate workers [13]. Researchers have carried out many malaria-related knowledge, attitude and practice (KAP) studies. Most KAP investigations have revealed low engagement in prevention practices among nearly all populations at risk for malaria [12, 14, 15]. According to Ming, malaria-related KAP among Chinese travellers exposed to malaria is far from satisfactory. That author suggested development of specific educational tools to reduce the rate of malaria infection [16]. With respect to the relationship between knowledge and attitude, in which behavioural change is not always direct and positive, health literacy has emerged as an independent research field with the potential to further explain such complex relationships [17]. Health literacy is an achieved level of knowledge or proficiency that depends upon an individual’s capacity and the resources provided by the health care system [18]. Investigations have elucidated the prevalence of health literacy in relation to a population’s health knowledge, health behaviours and health outcomes [19–21]. Education is an effective strategy for changing health literacy and thereby changing behavioural risk factors, as well as for reducing unfavourable conditions and associated diseases [22, 23]. Most Chinese companies, particularly petro-oil companies located in Africa, have set up strategies for the prevention and treatment of malaria among non-immune travellers and expatriate workers by following the OGP/IPIECA Malaria Management Programme [9]. Compliance with these programmes depends on health literacy [15]. To help with understanding how these strategies reach the population, assessing the malaria health literacy level can help to identify the main determinants that influence protective behaviours, and thereby assist in monitoring and evaluating progress made in the efforts to prevent and treat malaria among non-immune travellers and expatriate workers.

With the development of information technology (IT), social media and smartphones have become new channels and tools for information acquisition and exchange, and these are widely used in health information research [24]. There have been several studies of health education, behavioural improvement and disease treatment among community residents, women and populations in low-income countries [25–29]. According to the information-motivation-behaviour skills (IMB) model, information can be translated into action that can motivate individuals and eventually influence their attitudes and behaviour [30, 31]. Nearly all studies have found positive effectiveness of mobile or smartphone applications (apps) for health management [32, 33]. One review also confirmed that mobile technology interventions significantly improved an array of health care outcomes in Chinese populations [34–37]. Similarly, social networks such as Facebook, Twitter, YouTube, and WeChat provide new indexes in building relationships, finding and sharing information, and working co-operatively with others [38]. A systematic review proved the feasibility of delivering eHealth interventions to improve health literacy skills among people with different health conditions, risk factors and socio-economic backgrounds [39]. According to Korda and Itani, social media may be an effective platform with potential applications in the health communications field [40]. Researchers have provided health education via Facebook, which has led to positive health promotion results and encouraged others to build Facebook-based health education platforms and programs [41]. WeChat is one of the most popular social media platforms worldwide, with more than 0.54 billion users in more than 200 countries. Just as with other popular social networks, WeChat offers a free instant messaging application for smartphones that enables the exchange of voice, text, pictures, videos, and location information via mobile phone indexes [24, 42–44]. The present study is based on a new functional module of WeChat called WeChat official accounts, which can be freely acquired and used by companies to disseminate information and for internal communications. Individuals can read messages and communicate with others via these official accounts. The aims of this study were: (1) to investigate the malaria health literacy level of Chinese expatriates in Niger; and, (2) to develop a health education programme for the prevention and treatment of malaria among non-immune travellers and expatriate workers using WeChat official accounts, and evaluate its user satisfaction when applied to improving malaria health literacy among Chinese workers in Niger.

## Methods

### Study design and sample

This study was developed as part of the Niger Project of the China National Oil and Gas Exploration and Development Corporation (CNODC) in the towns of Niamey and Zinder, located in southeast Niger. An experimental design was used to evaluate the effectiveness of using WeChat official accounts for health education to improve malaria health literacy among Chinese expatriates in Niger. All staff members of the Niger branch of PetroChina Company Limited were selected to participate in the study. The inclusion criteria were as follows: (1) Chinese expatriates who would continue to work in Niger for ≥4 months; (2) those willing to participate in the study; and, (3) those familiar with using a WeChat official account to obtain information and communicate with others. Those who had to leave Niger within 4 months, who could not participate for the entire study period of 4 months, who refused to participate or were unwilling to use the WeChat platform were excluded. A total of 1441 participants who met the inclusion criteria were included in the study.

Before the intervention began, all participants were asked to complete a baseline malaria health literacy questionnaire that was administered by onsite physicians and health, safety, and environment (HSE) managers. A random number table was used to randomly divide participants into two groups according to their working location area number. There were 721 participants in the intervention group and 720 in the control group. A malaria-related health education programme was developed and during the period between 1 July and 30 October, 2014 sent a total of 50 messages (three messages per week, on average) to participants in the intervention group. At the same time, news were sent health-related news to participants in the control group. Messaging technology was used to concurrently send different messages to different target populations. Participants in the intervention group could also ask malaria-related health questions on the ‘Healthy Family’ official account consultation page at any time during the 4-month period. Participants in both groups maintained the routine strategies for prevention and treatment of malaria in non-immune travellers and expatriate workers stipulated by the HSE management system of the CNODC Niger project.

**Fig. 1:**
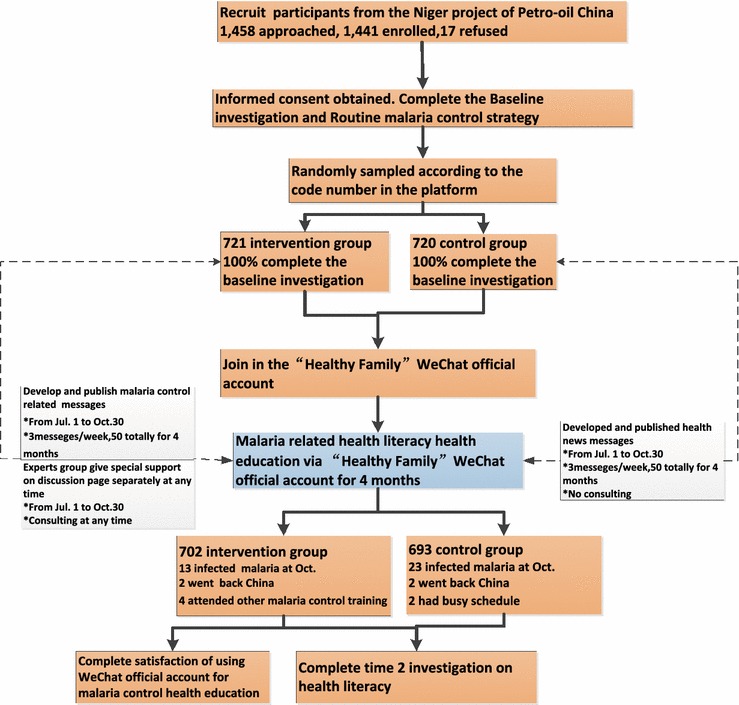
Study protocol flowchart of malaria health education via WeChat official account

Health literacy questionnaires were administered twice, before and after the education intervention. In addition, participants in the intervention group were subsequently queried about their satisfaction with using the WeChat official account as a malaria prevention education strategy. Data of malaria morbidity rates during the study period for participants in both groups were also collected and compared. These data were used as the baseline and endpoint of the investigation. The study flowchart is shown in Fig. 1.

### Malaria health literacy questionnaire development

The historical data, health literacy-related theories, relevant research papers, WHO recommendations, OGP/IPIECA guidelines for malaria prevention, and experiences of onsite health management were reviewed to assist in developing a first draft of the malaria health literacy questionnaire. The Delphi method, pilot onsite testing and validation processes [45] were used to develop and evaluate the reliability and validity of the questionnaire. After two rounds of expert review and modification, the final version of the questionnaire proved reliable and valid [46]. There were four aspects, 15 indexes and 46 questions addressing the malaria health literacy of Chinese expatriates (Table 1). The final questionnaire comprised the following content: knowledge related to the prevention and treatment of malaria in non-immune travellers and expatriate workers (four indexes, 16 questions) including knowledge of the definition, prevention, diagnosis, and treatment of malaria; attitudes towards the prevention and treatment of malaria in non-immune travellers and expatriate workers (three indexes, eight questions) including attitudes about knowledge acquired about malaria prevention and treatment among non-immune travellers and expatriate workers and about timely treatment; practices related to prevention and treatment of malaria in non-immune travellers and expatriate workers (four indexes, 14 questions) including practices of information acquisition, malaria prevention, helping others, and malaria diagnosis and treatment; and lastly, skills with respect to the prevention and treatment of malaria among non-immune l and expatriate workers (four indexes, eight questions), mainly skills in the use of rapid diagnostic tests (RDTs) and results interpretation as well as correct treatment dosage according to a doctor’s advice.

**Table 1:** Content of the malaria health literacy questionnaire

Aspects	Indexes	Question number	Total question
Knowledge	Malaria transmission knowledge	4	
	Malaria prevention related knowledge	5	
	Malaria diagnosis standard	4	
	Malaria treatment approach	3	
			16
Attitude	Malaria prevention attitude	2	
	Wish to acquire malaria information	4	
	Malaria diagnosis and treat on time	2	
			8
Practice	Acquire malaria information activity	3	
	Comprehensive malaria prevention practice activity	6	
	Help others activity	2	
	Malaria diagnosis and treatment practice	3	
			14
Skill	Knowledge acquire skill self-assessment	2	
	Malaria diagnosis skill self-assessment	2	
	Using RDT skill	2	
	Reading doctor’s advice skill	2	
			8

#### Notes

Scores for each question were as follows [46, 47]: 1 point scored for correctly answered single-choice questions and completion questions; 1 point scored when choosing ≥60% correct answers on multiple-choice questions. Each question had one score, so the total possible score was 46 points for malaria health literacy. Scores ≥70% of the total and each aspect was considered to be required. Scores for each aspect of malaria health literacy were as follows: 32 for the malaria health literacy (total possible score of 46), 11 for the knowledge aspect (total possible score of 16), 6 for the attitude and skills aspects (total possible score of 8 each), and 10 for the practice aspect (total possible score of 14) and 6 for the skill aspect (total possible score of 8).

### Data collection

#### Demographic data

General demographic data was collected, which including age, total number of years worked, time working in Niger, education level, malaria history, self-assessed health status, and malaria-related health education history and practice. The baseline malaria health literacy questionnaire was administered at the same time.

#### Malaria morbidity data

The CNODC infectious disease network reporting system was used to track the number of people infected with malaria who were working at the Niger project. Two qualified onsite professionals performed microscopic examination and RDT in suspected malaria cases.

#### Participant satisfaction data

Participant satisfaction was assessed using a revised mHealth satisfaction survey [48]. The satisfaction survey was administered only to participants in the intervention group, together with the post-intervention malaria health literacy questionnaire. The satisfaction survey comprised Likert-scale questions assessing various aspects of the programme including perceived efficacy, opinions about the types and frequency of messages, appropriateness of the level of message content, and willingness to continue with or recommend the programme to travellers or new arrivals.

### Data analysis

Data analysis was conducted using IBM SPSS, Version 19 (IBM Corp, Armonk, NY, USA). Four sets of analyses were performed. First, simple Chi squared statistics and *t* tests were used to test statistical differences in the demographic characteristics and malaria health literacy between the health education intervention group (WeChat official account prevention and treatment of malaria among non-immune travellers and expatriate workers) and the control group. Second, Chi squared analyses were used to test differences in malaria health literacy between the two groups. Third, Chi squared statistics were applied to compare the number of malaria infections between the two groups from July to October, 2014. Fourth, descriptive statistics were used to analyse satisfaction with using the WeChat official account health education programme among participants in the intervention group. *p* < 0.05 was considered statistically significant.

### WeChat malaria health education programme development

**Fig. 2:**
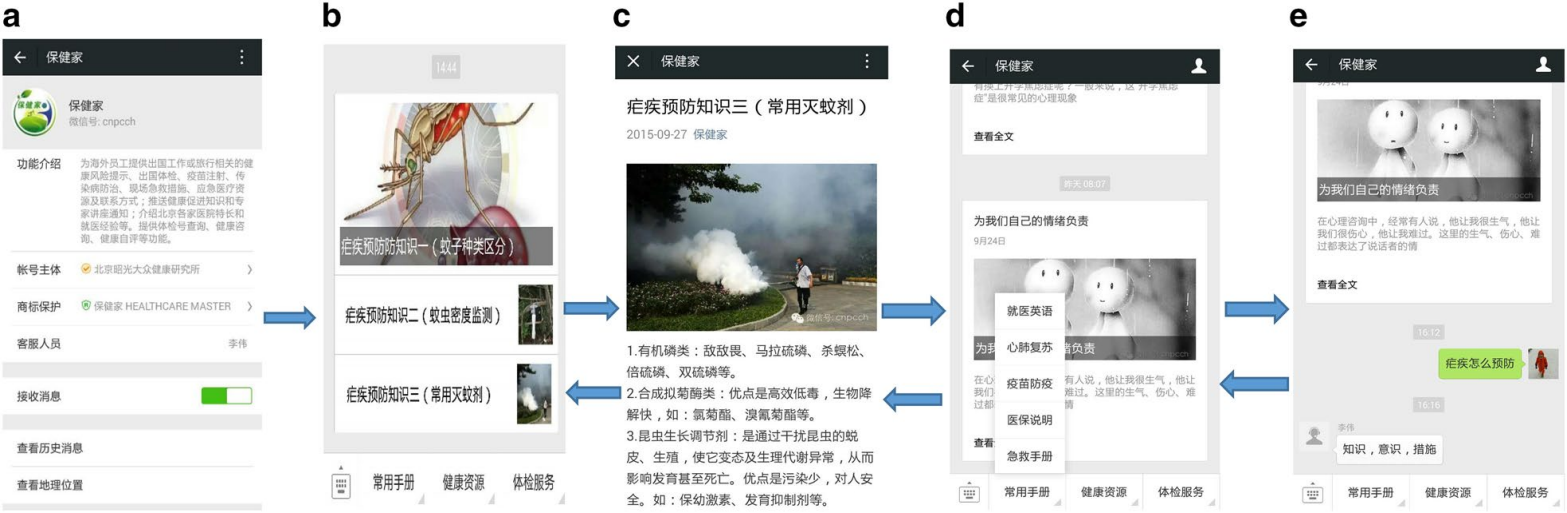
Application flow sheet of malaria control health education in the ‘Healthy Family’ WeChat official account. **a** Profile of the ‘Healthy Family’ WeChat official account; **b** profile of the message sending page; **c** profile of the message reading page for staff; **d** profile of history message review page for staff; **e** profile of the consulting page for staff with experts

A WeChat official account can be used to deliver health education messages related to prevention and treatment of malaria in non-immune travellers and expatriate workers using a special feature for delivering the contents of learning modules and to provide discussion forums. The HSE department of the CNODC developed the Healthy Family WeChat official account with the technological support of a specialized IT company. The health education programme was developed for use with the Healthy Family WeChat official account (Fig. 2a). All participants were asked to follow the official account after completing the baseline survey. Both participant groups could read new messages (Fig. 2b, c) and review the message history (Fig. 2d) of content published in the official account. Messages sent to the intervention group only comprised content related to information about the prevention and treatment of malaria among non-immune travellers and expatriate workers; messages sent to the control group only contained health-related news. A consultation functional interface (Fig. 2e) was open only to the intervention group, for communication with health experts. Six experts (two from Beijing Ditan Hospital specializing in prevention and treatment of malaria in non-immune travellers and expatriate workers, two health management specialists from the HSE department of the CNODC, and two onsite doctors from the PetroChina Company Hospital) made up the consulting group, which was formed to individually address questions from participants in the intervention group on the prevention and treatment of malaria in non-immune travellers and expatriate workers. Participant questions were only visible to members of the expert panel, and experts were asked to answer queries within 24 h.

Development of the WeChat official account used for the health education programme was supported by a health education expert panel that included two professors from Peking University Health Science Centre specialized in health education; three doctors from Beijing Ditan Hospital whose specialty was malaria prevention and treatment; and two researchers from the Chinese Centres for Disease Control and Prevention, experts in epidemiological investigation and prevention and treatment of malaria in non-immune travellers and expatriate workers. The entire research team participated in the design and drafting of messages, and all messages were evaluated by the expert team to ensure their accuracy, acceptability and comprehensibility.

All malaria prevention and treatment health education messages were created based on WHO recommendations, the OGP/IPIECP guidelines, published literature, onsite health management policies, and results of the baseline health literacy survey. There were four phases in development of the Healthy Family WeChat official account messages [49]: (1) a research group meeting was held each week to identify three detailed content messages that would be published in the health education programme the following week; (2) each message was compiled by one research group member who was familiar with the topic. This expert researched and developed appropriate multimedia content using photos, clip art, interactive questions, or video clips to effectively convey the meaning of each message; (3) the text message and multimedia material were combined into one message, which was then revised according to expert opinion and subsequently approved by at least three experts in different domains; and, (4) one research team member was selected to be responsible for publishing messages in the Healthy Family official account on Mondays, Wednesdays and Fridays.

## Ethical considerations

The study protocol received ethical approval from the research and ethics committee of Peking University Health Science Centre. Oral consent was obtained from each participant prior to interviews. Participants’ names were not recorded; identification numbers were used instead. All information was treated confidentially and was only available to those directly involved with this research.

## Results

### General information

A total 1441 employees participated in the present study. Of these, 1398 (97.02%) were men; therefore, gender was not analysed separately in this study. Respondents’ age ranged from 21 to 54 years, with an average age 39.01 ± 7.02 years. Respondents’ time working in Niger ranged from 1 month to 11 years, with an average of 3.23 ± 1.99 years. Most participants had worked a total of more than 20 years (45.04%), had completed junior college (52.53%), and were employed in manual labour (95.00%). Most participants reported good or normal health (89.14%). A total 22.55% of the population self-reported a history of malaria. There were no significant differences between the WeChat official account education intervention group and the control group with respect to sociodemographic characteristics. All participants had previously received some type of malaria-related health training (Table 2).

**Table 2:** Sociodemographic characteristics of Chinese expatriates in Niger: WeChat official account intervention group versus control group

Contents	Group	Baseline total (n = 1441)		Intervention group (n = 721)		Control group (n = 720)		χ^2^	p
		n	%	n	%	n	%		
Age (years)	20–<30	216	14.99	106	14.70	110	15.28	2.311	0.510
	30–<40	493	34.21	253	35.09	240	33.33		
	40–<50	690	47.88	337	46.74	353	49.03		
	50–≤60	42	2.91	25	3.47	17	2.36		
Education	Junior college	757	52.53	385	53.40	372	51.67	0.467	0.792
Level	Under-graduate	588	40.80	288	39.94	300	41.67		
	Post-graduate	96	6.66	48	6.66	48	6.67		
Occupation	Manuel labor	1369	95.00	680	94.31	689	95.69	1.447	0.229
	Manager	72	5.00	41	5.69	31	4.31		
Working period	0–≤10	396	27.48	196	27.18	200	27.78	0.937	0.816
	11–<21	396	27.48	195	27.05	201	27.92		
	21–<31	619	42.96	316	43.83	303	42.08		
	≥31	30	2.08	13	1.80	17	2.36	2.362	0.307
Work in Niger	<3	846	58.71	409	56.73	437	60.69		
	3–<5	556	38.58	292	40.50	264	36.67		
	≥5	39	2.71	20	2.77	19	2.64		
Health condition	Good	486	33.73	243	33.70	243	33.75	0.742	0.690
Self-assessment	Normal	799	55.45	395	54.79	404	56.11		
	Bad	156	10.83	83	11.51	73	10.14		
Malaria history	Yes	1116	77.45	550	76.28	566	78.61	1.118	0.290
	No	325	22.55	171	23.72	154	21.39		
Malaria education	Yes	1441	100	721	100	720	100		
	No	0	0	0	0	0	0		

### Malaria health literacy of respondents

**Fig. 3:**
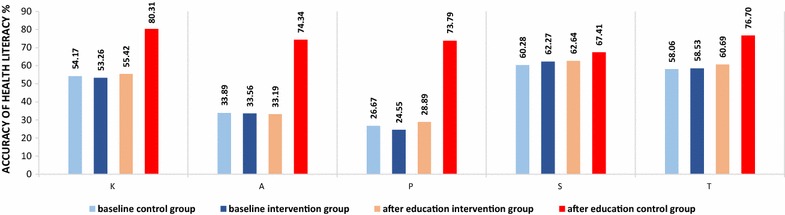
Comparison of changes in malaria-related health literacy accuracy rate between the intervention and control groups

The malaria-related knowledge of participants is summarized in Table 3 and Fig. 3. Before the WeChat official account health education intervention, participants’ overall health literacy was approximately 58.29, 58.53 and 58.06% for the intervention group, control group and total population, respectively. The highest literacy score (approximately 62%) was for skills, both for the total and for each group; this was followed by the score for knowledge (approximately 54%). However, with respect to skills, only 25% of participants used RDTs correctly. Participants had less knowledge about comprehensive prevention strategies (approximately 13%). The results showed low attitude scores for the total population (approximately 34%). Practice scores presented the lowest accuracy rate of the total (about 25%), especially for information acquisition and multiple malaria prevention practices, with accuracy rates only around 18 and 16%, respectively.

**Table 3:** Comparison of accuracy rate of malaria-related health literacy and major indexes between the two groups, before and after intervention

Aspect	Indexes	Before intervention				χ^2^	p	After intervention				χ^2^	p
		Intervention		Control				Intervention		Control			
		n	%	n	%			n	%	n	%		
Knowledge	Malaria transmission knowledge	400	55.48	386	53.61	0.057	0.477	607	84.19	399	55.42	141.5	<0.001
	Malaria prevention related knowledge	345	47.85	327	45.42	0.857	0.355	593	82.25	348	48.33	182.9	<0.001
	Malaria diagnosis standard	100	13.87	99	13.75	0.004	0.948	570	79.06	111	15.42	585.4	<0.001
	Malaria treatment approach	633	87.79	640	88.89	0.419	0.518	650	90.15	639	88.75	0.751	0.27
Attitude	Malaria prevention attitude	426	59.08	427	59.31	0.007	0.932	611	84.74	431	59.86	111.42	<0.001
	Wish to acquire malaria information	664	92.09	669	92.92	0.351	0.553	678	94.04	659	91.53	4.115	0.042
	Malaria diagnosis and treat on time	377	52.29	373	51.81	0.034	0.854	588	81.55	380	52.78	133.98	<0.001
Practice	Acquire malaria information activity	131	18.17	133	18.47	0.022	0.882	663	91.96	150	20.83	738.24	<0.001
	Comprehensive malaria prevention practice activity	122	16.92	133	18.47	0.595	0.440	508	70.46	140	19.44	376.48	<0.001
	Help others activity	669	92.79	676	93.89	0.702	0.402	681	94.45	652	90.56	8.96	0.02
	Malaria diagnosis and treatment practice	715	99.17	714	99.17	0.000	0.998	711	98.61	710	98.61	0.00	0.998
Skill	Knowledge acquire skill self-assessment	565	78.36	564	78.33	0.000	0.989	602	83.50	571	79.31	4.177	0.041
	Malaria diagnosis skill self-assessment	605	83.91	614	85.28	0.516	0.472	616	85.44	600	83.33	1.21	0.271
	Using RDT skill	183	25.38	183	25.42	0.000	0.998	587	81.41	188	26.11	443.32	<0.001
	Reading doctor’s advice skill	405	56.17	411	57.08	0.122	0.727	568	78.78	417	57.92	72.49	<0.001

### Effectiveness of the WeChat official account health education intervention strategy for prevention and treatment of malaria in non-immune travellers and expatriate workers

There were improvements in the malaria health literacy of participants in both the intervention and control groups after the WeChat official account health education intervention (Table 3; Fig. 3). There was a greater change in KAP, skills and overall health literacy among the intervention group compared with the control group, and this difference was statistically significant (*p* < 0.01). This was especially true for malaria-related knowledge acquisition, practices, and attitude, as well as comprehensive intervention practices and correct use of RDTs (*p* < 0.001). After the intervention, all aspects of health literacy showed accuracy rates above 70%. Figure 3 compares changes in the accuracy rate of malaria health literacy scores between the intervention and control groups.

 In the intervention group, the reported malaria morbidity among participants from July to October 2014 decreased from 23.72 to 15.40%; that of the control group showed less change. There were significant differences between the intervention and control groups with respect to malaria morbidity (*p* < 0.05). There were also statistically significant differences in malaria morbidity for the intervention group between 2013 and 2014 (*p* < 0.05). Table 4 shows these changes.

**Table 4:** Comparison of malaria morbidity in the two groups from July to October, 2013 and 2014

2013		χ^2^	*p*	2014		χ^2^	*p*	χ^2^*	p*	χ^2#^	*p* ^#^
Intervention, N = 721	Control, N = 720			Intervention, N = 721	Control, N = 720						
152	159	0.213	0.644	111	142	4.660	0.018	7.817	0.03	1.214	0.150

χ^2^* and p* were the results of comparison of the intervention group between 2013 and 2014

χ^2#^ and *p* ^#^ were the results of comparison of the control group between 2013 and 2014

### Satisfaction with the WeChat official account health education intervention strategy for prevention and treatment of malaria in non-immune travellers and expatriate workers

Overall, participants reported high levels of satisfaction with the WeChat health education official account. Specifically, 82.80 and 83.35% of participants indicated they would personally like to continue with the educational programme and health consultations, respectively, and 100% would recommend the programme to family or friends. Similarly, more than 90% of participants agreed that education via WeChat official accounts was an effective way of communicating, that the education messages were understandable, and that they enjoyed receiving them frequently each week, especially during the rainy season. Table 5 gives further details of participant responses regarding user satisfaction.

**Table 5:** Evaluation of prevention and treatment of malaria in nonimmune travelers and expatriate workers health education program via “Healthy Family” WeChat official account intervention group at 4 months (n = 721)

Satisfaction assessment questions	Strongly agree	Agree	Neutral	Disagree	Strongly disagree
	n (%)	n (%)	n (%)	n (%)	n (%)
Using WeChat official account is a good way to teach me about malaria	332 (46.05)	367 (50.90)	15 (2.01)	7 (0.97)	0
I have enjoyed the “Healthy Family” official account	317 (43.97)	368 (51.04)	27 (3.74)	9 (1.25)	0
I was able to understand the all the messages	381 (52.84)	306 (42.44)	20 (2.77)	14 (1.94)	0
The new messages came at times that were good for me	301 (41.74)	401 (55.62)	11 (15.26)	7 (0.97)	0
I was motivated by the prevention and treatment of malaria in nonimmune travelers and expatriate workers challenges	197 (27.32)	412 (57.14)	112 (15.53)	69 (9.57)	0
The messages help me and remind me to protect myself from getting malaria	372 (51.60)	296 (41.05)	35 (4.85)	18 (2.50)	0
I like the consulting function of the WeChat official account	297 (41.19)	401 (55.62)	20 (2.77)	3 (0.42)	0

	Too basic n (%)	About right n (%)	Too hard/complicated n (%)
The information in the messages	7 (0.97)	672 (93.20)	42 (5.83)
The consulting information provided by experts	9 (1.25)	681 (94.45)	31 (4.23)

	Too few n (%)	About right n (%)	To many n (%)
The number of messages each week	33 (4.58)	673 (93.34)	15 (2.08)

	Less n (%)	Stay the same n (%)	More n (%)
If the program were to continue, I like the number of weekly messages to be	45 (6.24)	517 (71.71)	159 (22.05)

	No help n (%)	About right n (%)	Very helpful n (%)
The consulting function of the WeChat official account	31 (4.30)	591 (81.97)	99 (13.73)

	Yes n (%)	No n (%)
I would like to continue receiving messages about malaria	597 (82.80)	124 (17.20)
I would like to continue using the consulting function	601 (83.35)	120 (16.64)
I would recommend this official account to a travelers or new arrivals	721 (100)	0

## Discussion

International long-term travellers and labourers working abroad who are from non-endemic areas are populations at high risk of malaria because they lack immunity. Malaria is one of the leading causes of mortality and morbidity in developing countries, particularly in sub-Saharan regions such as Niger, where the disease is very common. Expatriates working in these high-burden countries are often located in remote rural areas, with poor access to advanced medical care. Distance and safety are two barriers to malaria professionals and health managers being able to provide continuous or even timely onsite medical support. Many studies have found significant morbidity and mortality among expatriate populations owing to malaria [7, 8], and lower KAP levels for prevention and treatment of malaria among non-immune travellers and expatriate workers [14]. Previous studies on health education strategies for malaria prevention have proved to be effective in improving malaria prevention KAP and decreasing rates of malaria morbidity [50, 51]. There is little research on malaria KAP and prevention strategies among expatriates, despite being a high-risk population. Health literacy is often used to evaluate the degree to which individuals have the capacity to obtain, process and understand basic health information and services so as to make appropriate health decisions. Low health literacy is related to poor health practices and outcomes [20]. Understanding and measuring health literacy in relation to behavioural risk factors is an important goal in the prevention, detection and management of disease [39]. It is widely accepted that mHealth strategies have the potential to be used as an educational tool for behavioural change. mHealth interventions have enormous potential to help control the spread of epidemics and thus reduce associated deaths; therefore, such strategies should be further exploited [52]. As popular as Facebook and Twitter are among Chinese people worldwide, the WeChat application is a social media tool that can act as a bridge to provide health support through the dissemination and communication of information across great distances. Information related to the prevention and treatment of malaria in non-immune travellers and expatriate workers can be acquired and requested at any time via such applications, whose technology enables connection and communication between professionals and individuals. This study aimed to identify the malaria health literacy level of Chinese expatriates working in Niger and evaluate the effectiveness and feasibility of the WeChat mobile app for health education.

Results of the study showed that malaria health literacy among Chinese expatriates is not high (<60%), especially for malaria prevention attitudes and practices (both below 35%). This is very similar to the results of other malaria-related KAP studies [14, 15]. The following possible factors should be considered [53]: (1) Niger is a tropical region where, because of the hot weather, some people may feel uncomfortable sleeping under a bed net or wearing long-sleeved clothing; (2) traditional Chinese lifestyle habits, such as exercising outdoors in the morning or evening and opening windows for ventilation, may be dangerous behaviours in malaria-risk areas. Such dangers are often ignored by expatriates, who find it difficult to change behaviours; (3) effective malaria prevention strategies, such as insecticide-treated bed nets (ITNs) and chemoprophylaxis, are considered harmful to health or are unavailable [54] to some people. In this study, some respondents stated they had no way of obtaining detailed information about these prevention strategies; (4) malaria is the most common disease in Niger, but it is seldom seen in China. Therefore, Chinese expatriates have difficulty understanding the level of risk and potential severity of the disease; (5) although health education regarding prevention and treatment of malaria in non-immune travellers and expatriate workers is required by the CNODC HSE department for each employee working in Niger, most workers cannot absorb such large amounts of knowledge in a short time, and much of the information provided is soon forgotten; (6) in this study, Chinese expatriates working on the Niger project had little knowledge about malaria diagnosis and progression. Therefore, although RDT skills are not difficult to learn, participants did not consider this a vital skill for onsite staff, and only 25% of participants could use RDTs properly; and, (7) limited information is another barrier for staff to acquire useful health knowledge when needed or when faced with problems in remote rural areas. This study established that health managers and local doctors must give staff incentives and conduct regular long-term training to help them obtain malaria-related knowledge and connect with malaria professionals whenever necessary.

The messages on the Healthy Family WeChat official account developed in this study were based on the results of onsite health literacy investigation, literature review, local conditions, and the advice of malaria professionals. Text messages, photos, voice, and video were all used in developing the health education programme. These types of messages may attract more interest and willingness to engage among users. For example, compared with microscopy, RDT diagnosis of malaria in patients with uncomplicated febrile illness has high effectiveness and low cost [55]. How-to-use steps for RDT and how to read the results were shown in photographs. So that staff could easily follow in the intervention. DEET has been reported to be an effective repellent for malaria vector control that offers 96% protection and 8 h protection [54]. Researchers have suggested that educational programmes should increase knowledge and awareness about the use of indoor insecticide to prevent malaria [56]. Video materials were developed to explain how to obtain and use DEET in Niger for staff as a reference whenever they need. Individual, ecological and environmental risk factors are regarded as targets of control efforts to achieve further reduction and elimination of malaria [57, 58]. Messages on the infection process of malaria under various conditions were explained via the intervention using onsite photographs to stimulate greater interest among staff in changing risky behaviours and habits. All messages could be reviewed at any time in the message history review profile of the WeChat account.

There are over 10,000 labourers working in Africa and malaria is one of the most severe health risks for these workers. WeChat official accounts provide a new means of propagating information using IT technology and frequent multimedia messages, for better communication and health management among users. The Healthy Family official account developed by the CNODC HSE department aims to disseminate health-related information to company staff worldwide. Lecture training materials and handbooks on malaria prevention are provided to each employee prior to leaving for Africa, but this is inefficient and costly. Chou et al. noted “opportunities for narrowing the health disparities gap exist through effective use of social media as communication and health promotion platforms” [59]. In this study, implementing a health education programme for prevention and treatment of malaria in non-immune travellers and expatriate workers via WeChat official accounts achieved good results in improving the health literacy of expatriates in Niger, as well as in lowering the malaria infection rate. WeChat can therefore be considered an important health promotion tool. One systematic review of eHealth interventions specifically designed to improve the health literacy skills of people with different health conditions and risk factors [39] confirmed that using Facebook for online nutritional education among low-income women [60], as well as message-based mobile health interventions, improves clinical outcomes and increase healthy behaviours [61]. In the context of encouraging interest in health care information via social media such as WeChat and Facebook, users can communicate with healthcare providers with no limitations of time or location. Healthcare providers can enrich multimedia content using engaging narration, text, graphics, sound, and video presentations [60], as in this study. Health managers should coordinate with hospitals, disease control centres, and other professional health-related agencies, to implement current advanced technologies in health communication and promote important healthcare information as broadly as possible [60].

Eliminating malaria is the common goal of WHO and many nations worldwide, including China. This study developed an effective, feasible and acceptable malaria health education and health management tool using WeChat official accounts for expatriates working in remote areas at high risk for malaria, which can be used as a reference for similar health management programmes.

## Conclusions

Malaria health literacy among Chinese expatriates in Niger is not high. The present health education intervention for the prevention and treatment of malaria in non-immune travellers and expatriate workers via WeChat official accounts proved to be effective, sustainable, feasible, and well accepted as a means to improve the malaria health literacy of the expatriate study population. This health education intervention strategy can be used as a reference for local malaria elimination and health promotion programmes.
